# Supplementary material for: Patterns of Intron Gain and Loss in Fungi
Source: PLoS Biol. 2004 Nov 30;2(12):e422. doi: 10.1371/journal.pbio.0020422 (PMC532390; doi:10.1371/journal.pbio.0020422)
Supplement: Table S1 — Also available at http://genes.mit.edu/NielsenEtAl/. (4.3 MB ZIP). [file pbio.0020422.st001.zip › NielsenEtAl/html/944.html]

AN5523.1.NCU09715.1.MG03860.1.FG06051.1


```
 CLUSTAL W (1.82) Multiple Sequence Alignments - Introns Inserted


Sequence 1: NCU09715.1	523 aa
Sequence 2: MG03860.1	499 aa
Sequence 3: FG06051.1	603 aa
Sequence 4: AN5523.1	504 aa
Alignment Length: 603 aa
Number Identitical Residues: 360 aa
Alignment Score (without introns) 15535


MG03860.1 	---------------~----~---------------~-----------------------
NCU09715.1	---------------~----~---------------~-----------------------
FG06051.1 	MLVIERSDQIRWMSI1SRQE2HKLTRYVNVASSLLS2VIVLLLNNPKLLALSLTPLSTGD
AN5523.1  	---------------~----~---------------~-----------------------
          	                                                            

MG03860.1 	-----------~-MGSVEDETALPSGGRLLLISNRLPITIKRSDDGQYSFSMSSGGLVTG
NCU09715.1	-----------~-MPGVIEPTGS---GRLLLISNRLPITIKRADDGQYTFSMSSGGLVTG
FG06051.1 	LRLSALRQLPV1RMPETVGENGNAAPGRLLLLSNRLPITIKRSEDGSYSFSMSSGGLVTG
AN5523.1  	-----------~-MPGVEKSSQNE--SRLLLVSNRLPITIKRSEDGKYDFSMSSGGLVSG
          	             *  .   .     .****:**********::**.* *********:*

MG03860.1 	LSGLAKTTSFQWYGWPGLEVPDAEAGPVVQRLKNEYGAHPVFVDDELADRHYNGFAN1SI
NCU09715.1	LSGLAKSTKFQWYGWPGLEVPEAETAGMKQRLKEEYDAIPVFVDDELADRHYNGFAN1SI
FG06051.1 	LSGLSKTTSFQWYGWPGLEVPDNEVEGMKQRLKEEYGAHPVFIDDELADRHYNGFSN1SI
AN5523.1  	LSGLSKTTTFQWYGWPGLEVPEEEIPTLKNRLKEEYNAIPVFIDDELADRHYNGFSN1SI
          	****:*:*.************: *   : :***:**.* ***:************:* **

MG03860.1 	LWPLFHYHPGEITFDESAWSAYKEVNRLFAQTVVKDVQDGDMIWVHDYHLMLLPEMLREE
NCU09715.1	LWPLFHYHPGEITFDESAWAAYREVNRLFAKEVAKDVQDGDMVWVHDYHMMLLPQMLREE
FG06051.1 	LWPLFHYHPGEITFDESAWAAYQEVNRLFAKTVIKDVQDGDLIWVHDYHLMLLPQMLREE
AN5523.1  	LWPLFHYHPGEITFDESAWEAYKEANRLFAQAVASQVQDGDLIWVHDYHLMLLPEMLREE
          	******************* **:*.*****: * .:*****::******:****:*****

MG03860.1 	IGDSKKNVKIGFFLHTPFPSSEIYRILPVRQALLQGVLHCDLLGFHTYDYARHFLSSCSR
NCU09715.1	IGDTKKNVKIGFFLHTPFPSSEIYRILPVREALLLGILDCDLIGFHTYDYARHFLSSCSR
FG06051.1 	IGESKKNVKIGFFLHTPFPSSEIYRILPVREALLTGLLDCDLIGFHTYDYARHFLSSCSR
AN5523.1  	IGNTKKNIKIGFFLHTPFPSSEIYRILPVRNELLLGLLHCDLIGFHTYDYTRHFLSACSR
          	**::***:**********************: ** *:*.***:*******:*****:***

MG03860.1 	IL2SAPTTPNGVQFAGRFVTVGAFPIGIDPEKFVEGLQKPKVQQRIAALTRKFEGVKLIV
NCU09715.1	IL2QTPTTPNGVDYNGKFVTVGAFPIGIDPEKFVEGLKKQNVQQRIAALTRKFEGVKLIV
FG06051.1 	IL~ECPTTPNGVDWNGRFVTVGAFPIGIDPDNFVEGLKKPKVQERIAALSRKFEGVKLVV
AN5523.1  	LL2GLPTTPNGIEFQGKIIACGAFPIGIDPEKFKEGLKKEKVQKRIATLEQKFQGVKLMV
          	:*   ******::: *:::: *********::* ***:* :**:***:* :**:****:*

MG03860.1 	GVDRLDYIKGVPQKLHALEVFLTEHPEWIGKIVLVQVAVPSRQDVEEYQNLRAVVNELVG
NCU09715.1	GVDRLDYIKGVPQKLHALEVFLTEHPEWIGKIVLVQVAVPSRQDVEEYQNLRAVVNELVG
FG06051.1 	GVDRLDYIKGVPQKLHALEVFLTEHPEWIGKIVLVQVAVPSRQDVEEYQNLRAVVNELVG
AN5523.1  	GVDRLDYIKGVPQKLHALEVFLSDHPEWVGKVVLVQVAVPSRQDVEEYQNLRAVVNELVG
          	**********************::****:**:****************************

MG03860.1 	RINGKFG~TIEFMPIHFLHQSVSFDELAALYAVSDVCLVSSTRDGMNLVSYEYIATQRDR
NCU09715.1	RINGKFG~TVEFMPIHFLHQSVSFDELTALYAVSDVCLVSSTRDGMNLVSYEYIATQRER
FG06051.1 	RINGKFG~TIEFMPIHFLHQSVNFDELTALYAVSDVCLVSSTRDGMNLVSYEYIATQQKN
AN5523.1  	RINGKFG1TVEFMPIHFLHKSVNFDELIALYAVSDACVVSSTRDGMNLVSYEYIATQEKR
          	******* *:*********:**.**** *******.*:*******************...

MG03860.1 	HGVMILSEFTGAAQSLSGSLIVNPWNTEELANAIHDAVTMGPEQREANFKKLERYVFKYT
NCU09715.1	HGVMILSEFTGAAQSLNGSLIVNPWNTEELANAIHDAVTMSPEQREANYRKLERYVFKYT
FG06051.1 	HGVMILSEFTGAAQSLNGSLIVNPWNTEELANALHDAVTMSPEQREANYKKLERYVFKYT
AN5523.1  	HGSLVLSEFAGAAQSLNGSIIVNPWNTEELAAAYHEAVTMSDEQRALNFSKLDKYVNKYT
          	** ::****:******.**:*********** * *:****. ***  *: **::** ***

MG03860.1 	S~AWWGSSFVAELNRLAAGIEG-ADAE------------GSKTKIAIRG0----------
NCU09715.1	S~AWWGQSFVAELTRISAEAEKNANAQKEQHPIR--EGIANAANKAIEG~VTDALKSTNL
FG06051.1 	S~AWWGASFVAEMTRLSAEGSQPKTLRNVSGAVIGLEQKAQQIVTGLEK~KADELLSGNE
AN5523.1  	S2AFWGQSFVTELTRISEQAAG----------------KLPTKETPVNG~ETSKLETSSQ
          	* *:** ***:*:.*::                             :.   :.   : . 

MG03860.1 	------------
NCU09715.1	TGNQES------
FG06051.1 	KNETETSEEPSQ
AN5523.1  	------------
          	
```
